# Frozen elephant trunk: evolving techniques, persistent challenges, and the endovascular shift

**DOI:** 10.3389/fcvm.2025.1716491

**Published:** 2026-01-27

**Authors:** Muhammad Umar Nasir, Ali Yamani, Hamza Ghannam, Antonio Panza, Samuel Vester, Louis Louis, Cristiano Spadaccio

**Affiliations:** Department of Cardiac Surgery, University of Cincinnati College of Medicine, Cincinnati, OH, United States

**Keywords:** aorta-thoracic, endograft, endovascular aorta repair, frozen elephant trunk (FET), hybrid graft, meta - analysis, mini review

## Introduction

Open aortic arch replacement remains one of the most technically demanding operations in cardiovascular surgery, requiring careful orchestration of cerebral, myocardial, and visceral protection strategies (1, 2). The elephant trunk procedure's utility has been challenged over the last decade due to compounding risks of mortality of each subsequent procedure along with procedure interval mortality rate of approaching 11% (3). Also, several studies reported attrition rates as high as 50% owed to the long wait times between the 2 major procedures (3–5).

The advent of endovascular therapies such as TEVAR for high-risk patients led to the development of hybrid open-endovascular procedures for patients with extensive aortic disease (6, 7). Karck et al. in 2003 introduced “Frozen Elephant Trunk” (FET) technique, a hybrid procedure allowing a single-staged repair of arch and descending thoracic aortic disease. This innovation reduced interval mortality and loss-to-follow-up that affected the two-stage conventional elephant trunk (cET) approach (2, 8–12).

Over the past two decades, FET has evolved significantly, with refinements in cannulation, cerebral protection, and graft technology aimed at improving safety and expanding indications. Nevertheless, FET remains associated with specific risks, particularly spinal cord ischemia (SCI), distal stent-induced new entry, and bleeding (13). Simultaneously, the rapid emergence of branched and fenestrated stent-graft technology is reshaping the treatment algorithm, especially for high-risk or elderly patients (14).

The present review synthesizes and updates the latest evidence, including a meta-analysis of the most recent studies on FET, and discusses the evolving philosophy that increasingly integrates open, hybrid, and endovascular strategies.

### Commercially available devices used globally

**Figure 1:**
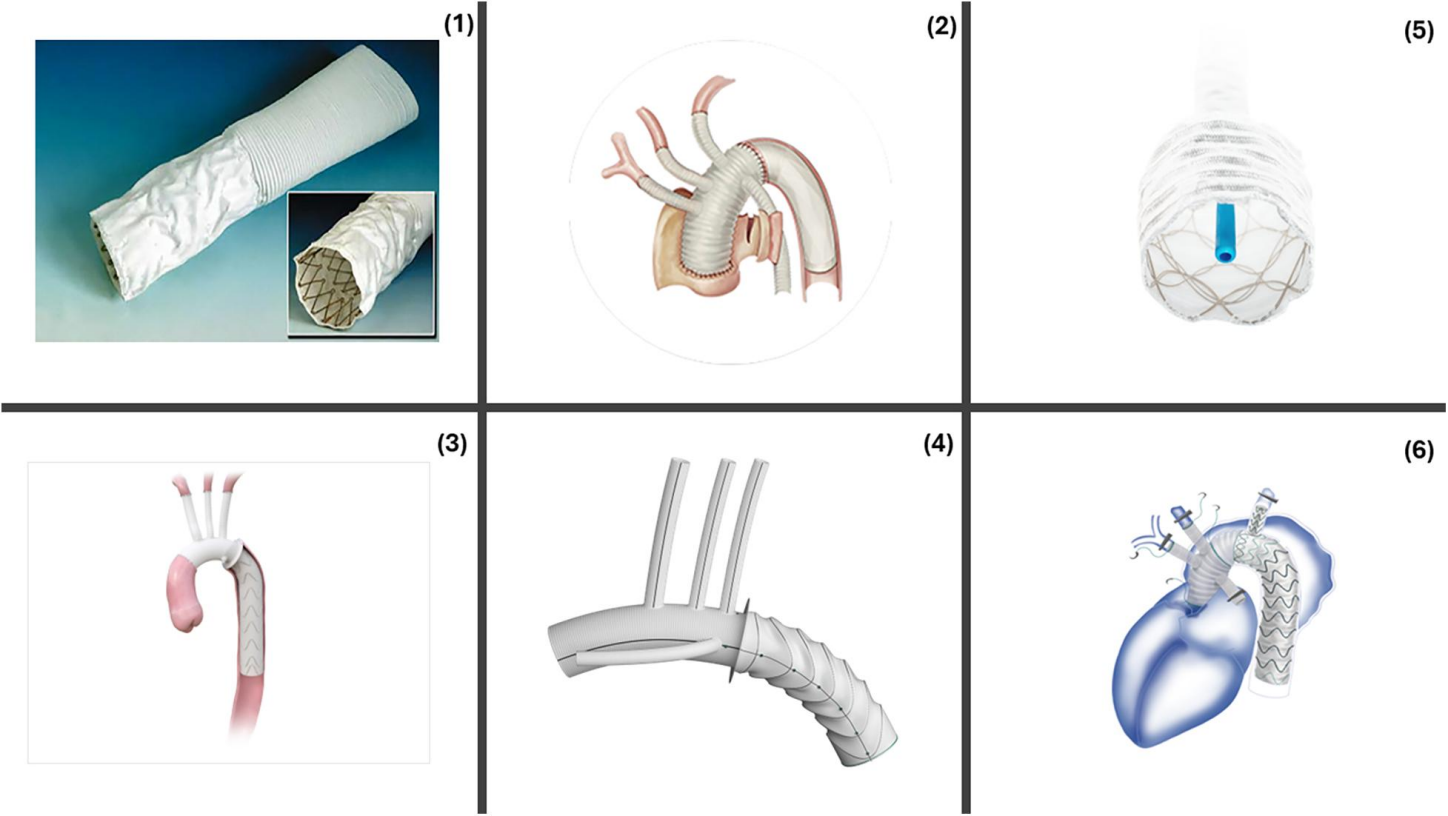
Commercially available FET devices. (1) Chavan-Haverich™ (Germany), the prototypical graft contains a stiff, large-diameter stent which limits its use in acute dissections. (2) Cronus™ (China) is a Co-Cr alloy. Its compactness allows for precise deployment **(A)** Before deployment **(B)** After deployment. (3) E-vita Open™ (Germany) includes a polyester graft with a flexible nitinol stent. E-vita Open Plus™ added a sewing collar to the earlier model allowing easier distal anastomosis. E-vita Neo™ added a tetra-furcate graft allowing anastomosis in more proximal zones, reduced SINE, precision deployment. (4) Thoraflex™ Hybrid includes four branches for arch vessels and a branch for perfusion. The circular nitinol ring arrangement helps minimize the radial force. (5) J Graft™ Frozenix has an oval, double-layered nitinol stents that accommodate the vessel curvature. (6) Fontus™ contains a branched stent-graft portion that allows zone 1 distal anastomosis, reduced circulatory arrest and simplified full arch reconstruction. Reproduced with permission from “The “hybrid prosthesis” (Chavan-Haverich endograft, Curative, Dresden, Germany) was made of a woven vascular prosthesis with stainless steel stents affixed to the inner aspects of its distal end” by Matthias Karck and Nawid Khaladj, licnesed under CC BY.

Several commercially available hybrid stent-graft devices developed globally since the advent of this procedure are briefly described in Figure 1.

## Technical considerations

### Cerebral perfusion strategies

Cerebral protection remains central to the safety of aortic arch surgery, regardless of the reconstructive technique employed. Antegrade cerebral perfusion (ACP) has become the preferred method over deep hypothermic circulatory arrest alone, as it allows for higher operative temperatures and shorter arrest times (15). A systematic review of randomized controlled and propensity-matched studies by Santos and colleagues demonstrated no consistent difference in mortality or permanent neurologic injury between unilateral and bilateral ACP, although the latter was associated with lower rates of transient neurologic dysfunction in some cohorts (16).

Bozso et al. further revealed that either strategy can achieve excellent outcomes with modern perfusion and operative techniques, supporting a safe, effective, and individualized approach to cerebral perfusion (2).

### Branch-first technique

Recently trifurcated graft techniques, enabling sequential head-vessel revascularization with continuous selective antegrade cerebral perfusion, have emerged as a means to balance cerebral protection with procedural complexity, and durability (17). Only 8.7% of patients in the cohort reported by Spielvogel et al. experienced adverse postoperative outcomes, also underscoring the safety of this technique (18). The trifurcated graft thus combines some of the advantages of separate reimplantation with the efficiency of a single modular construct.

Building on this concept, Matalanis and colleagues pioneered the branch-first technique, in which the supra-aortic vessels are reconstructed prior to circulatory arrest or deep hypothermia by sequentially anastomosing them to a branched graft under continuous cerebral perfusion (19–22). This allows for uninterrupted antegrade cerebral perfusion and avoids the ischemic burden associated with traditional arch repair. Technical reports highlight that perfusion can be maintained throughout using a trifurcated or multibranched graft, with subsequent distal arch and proximal ascending anastomoses performed under shorter (<30–40 min), more controlled circulatory intervals (23). Consistent with these physiological advantages, Abt et al. demonstrated in a multicenter study that a branch-first arch strategy is associated with lower perioperative mortality compared with conventional arch replacement techniques (24).

More recently, fenestrated frozen elephant trunk–based extra-anatomic bypass techniques have enabled supra-aortic vessel perfusion without arch reconstruction, potentially reducing neurologic complications and operative time (25, 26).

### Our approach

At our center, we have standardized a trifurcated branch-first strategy with continuous antegrade cerebral perfusion (tSACP) delivered via a simplified single-pump circuit. This approach avoids femoral cannulation—minimizing retrograde embolic risk in patients with acute dissection or heavy atheroma—and provides physiologic flow distribution among the three supra-aortic trunks (27). Because flow is distributed according to cerebral autoregulation and patient-specific vascular resistance, no flow adjustments are required during surgery. After cooling to moderate hypothermia (26–28 °C), we initiate cerebral perfusion through the trifurcated grafts, complete the distal anastomosis, and reimplant the head vessels sequentially, achieving near-continuous cerebral blood flow. This method has reduced lower-body ischemic time, enabled stable cerebral oximetry throughout the procedure, and yielded excellent outcomes in our 10-year experience (30-day mortality ≈8%, stroke ≈3%, SCI ≈2%) (28).

## Contemporary results of FET

### Meta-analysis: FET vs. cET

To enable a contemporary and methodologically robust comparison of FET and cET, we restricted our meta-analysis to comparative studies to reduce heterogeneity from unmatched populations and ensure that observed differences reflected operative strategy rather than case selection.

A comprehensive search of PubMed, MEDLINE, Scopus, and Web of Science was performed, covering the period from the introduction of the FET technique in 2003 through June 2025. The search strategy incorporated combinations of keywords such as “frozen elephant trunk,” “elephant trunk,” “arch replacement,” “hybrid arch,” “conventional elephant trunk,” and “FET vs. cET,” allowing for the identification of all potentially relevant adult surgical populations.

Studies were eligible if they involved adult aortic arch surgery with a direct comparison between FET and cET, including retrospective cohorts with clearly defined comparative designs and internal control groups. Studies were excluded if they were non-comparative, pediatric, non-original (such as reviews or meta-analyses), case reports, technical notes, or duplicates of previously published populations; in situations of overlapping cohorts, the larger or most comprehensive dataset was retained. After applying these criteria, fourteen studies encompassing a total of 3,125 patients met inclusion: 1,518 who underwent FET and 1,607 who underwent the conventional elephant trunk repair. The study selection process is summarized in the PRISMA flow diagram (Supplementary Figure).

Data extraction focused on 30-day mortality, in-hospital mortality, stroke, permanent neurologic deficit, spinal cord injury, and acute kidney injury. Extraction was performed independently by two reviewers, and discrepancies were resolved by consensus to minimize errors and subjective interpretation.

Given the retrospective nature of all included studies and the expected variation across centers in operative technique, perioperative management, and patient selection, all statistical analyses were conducted using a random-effects model based on the DerSimonian–Laird method. This approach accounts for both within-study and between-study variance and is considered the most appropriate model when heterogeneity—both clinical and methodological—is likely.

Effect sizes were calculated as relative risks with corresponding 95% confidence intervals. Statistical heterogeneity was evaluated using the I^2^ statistic, and values were interpreted according to established thresholds distinguishing low (I^2^ < 25%), moderate (I^2^ = 25%–50%), and high heterogeneity (I^2^ > 50%). Sensitivity analyses were performed using leave-one-out methodology to test the robustness of each pooled effect estimate and to ensure that no single study disproportionately influenced the results. When the number of available studies permitted, publication bias was explored through visual inspection of funnel plots supplemented by Egger's regression.

The risk of bias across the included studies was assessed using the Newcastle–Ottawa Scale (NOS), which evaluates three principal domains: the adequacy of cohort selection, comparability between groups, and the accuracy and completeness of outcome assessment. Total quality score 7–9 was considered good quality, 4–6 considered fair quality and 0–3 considered poor quality (Supplementary Table). The overall quality of the evidence was favorable, with the majority of studies scoring seven or more points, indicating generally low risk of selection or outcome bias despite the inherent limitations of retrospective observational data.

**Figure 2:**
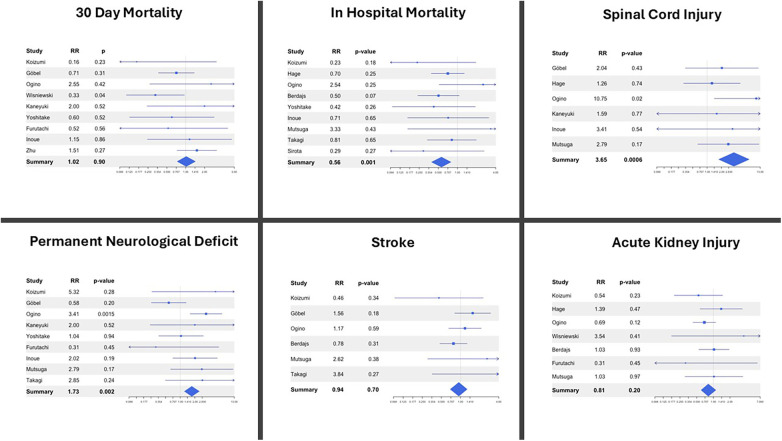
Forest plots describing post-operative outcomes between the frozen elephant trunk (FET) and conventional elephant trunk (cET) procedure. RR, relative risk.

FET was associated with a significantly lower in-hospital mortality compared with cET (RR 0.56; *p* = 0.001; I^2^ = 50%). However, this benefit was counterbalanced by an increase in spinal cord injury (RR 3.65; *p* = 0.0006; I^2^ = 0%) and a higher incidence of permanent neurologic deficit (RR 1.73; *p* = 0.002; I^2^ = 0%) in the FET cohorts. These findings are plausibly related to the physiological consequences of more extensive distal stent coverage and the associated reduction in intercostal perfusion, both of which increase vulnerability to spinal cord ischemia. However, although the length of the stented portion deployed in the descending thoracic aorta is recognized as one of the principal determinants of spinal cord injury, its specific contribution could not be evaluated in our meta-analysis due to the inconsistent and often incomplete reporting of stent length across the included studies. Notably, there were no significant differences between FET and cET in 30-day mortality, stroke, or acute kidney injury (Figure 2). Sensitivity analyses confirmed the stability of these findings, and heterogeneity was generally low to moderate across the pooled outcomes.

## Discussion

### The lessons learned: a practical guide

Even though the intervention of choice is guided by patient factors, disease etiology, and surgeon experience, increasing evidence suggests that strategies emphasizing early or continuous cerebral perfusion, such as trifurcated grafts and branch-first techniques, may offer superior safety profiles in contemporary practice (18).

Regardless of the approach, safety concerns persist due to the increased risk of postoperative spinal cord ischemia, a finding also evident in our meta-analysis of recent evidence. To enhance the safety and efficiency of FET implantation, several intraoperative strategies may be considered. A thorough review of computed tomography angiography (CTA) is essential to identify distal re-entry tears that could influence stent placement while prophylactic cerebrospinal fluid drainage should be employed to maintain spinal pressure below 12 mmHg (29, 30). Endoluminal inspection of the descending thoracic aorta before and after stent-graft deployment ensures accurate positioning and full expansion, and transesophageal echocardiography can further assist with delivery and confirmation of graft placement (31). Immediate resumption of systemic perfusion and rewarming following completion of the distal arch anastomosis is recommended to minimize ischemic time (32). Device selection must be tailored to pathology, with 10%–20% oversizing commonly applied in aneurysms, whereas true lumen–based sizing is preferred in dissections to avoid new entry tears (33). Stent length should be carefully chosen to minimize spinal cord ischemia, with preference for trunks shorter than 150 mm (29), and branched aortic arch grafts can be utilized when epiaortic vessels are involved to allow tailored revascularization (30). Finally, maintaining a mean arterial pressure above 80 mmHg optimizes spinal cord perfusion (34). Collectively, these technical nuances may help mitigate the risks of maldeployment, spinal cord injury, and distal stent-induced complications, while streamlining operative workflow in complex aortic cases.

### Disease-specific considerations

In chronic aneurysms and chronic dissections, the FET technique offers a more complete and durable single-stage repair of the aortic arch and proximal descending thoracic aorta, making it preferable to both stent-grafting and the conventional elephant trunk (cET) approach (35). However, its use is limited by a roughly 10% risk of spinal cord injury and persistent concern regarding incomplete false-lumen obliteration due to the associated stent-graft (36). In acute type A aortic dissection, FET provides the advantage of covering additional distal entry tears, allowing effective depressurization and promoting false-lumen thrombosis—whereas cET necessitates a distal surgical fenestration that maintains high false-lumen pressure (36). In acute type B dissection, FET may be considered when TEVAR is contraindicated, offering a less invasive alternative to open thoracic repair, although its use in this setting remains a Class IIb recommendation (30).

### The remaining challenges with FET

Antegrade stenting combined with open arch repair may improve long-term outcomes in DeBakey type I dissection, however its use remains controversial as it conflicts with the principle of minimizing procedural complexity and operative burden in acute dissection surgery (37, 38). Stent grafting in chronic aortic dissection remains controversial, as fibrotic and partially thrombosed aortic walls may prevent false-lumen obliteration and predispose to distal stent–induced new entry tears (dSINE) (39). If left untreated, dSINE has a mortality of up to 25% thereby requiring distal reintervention to prevent further dilation of the false lumen and eventual rupture (36, 40). The overall incidence of dSINE following FET in the literature is quite variable ranging from 0% to 27.3% (41). However, a recent meta-analysis consisting of 5,068 patients found a pooled estimate of 2% for dSINE after FET (40).

Concerns about an elevated risk of spinal cord ischemia have also caused significant debate in choosing the distal landing zone (42). Spinal cord ischemia was substantially linked to stent length >15 cm or coverage to or beyond T8, according to a recent meta-analysis of 35 trials including over 3,000 patients, potentially due to occlusion of intercostal arteries (29, 30). Interestingly, however, Takagi and colleagues claim that placing the FET at the T8 level can in fact, reduce distal malperfusion and spinal cord ischemia in patients with acute type A aortic dissection (43).

Interestingly, the Ascyrus Medical Dissection Stent is a hybrid arch graft that covers the descending aorta while allowing perfusion of the supra-aortic and intercostal vessels, potentially reducing hypothermia time, distal malperfusion, and spinal cord ischemia in type A aortic dissection (44).

### Emerging philosophies: endovascular and hybrid approaches

The success of endovascular abdominal aortic repair has driven the adoption of endovascular approaches in the thoracic aorta, shifting aortic arch repair paradigms and offering particular benefit to frail elderly patients who face higher mortality with open surgery (45).

A meta-analysis of 14 studies on physician-modified endovascular grafts in zone 2 landing zone revealed a technical success rate ranging from 83.3% to 100% in addition to left subclavian patency rates of 94% suggesting an alternative to the high-risk open repair. In addition, this study revealed a comparable 30-day mortality and spinal cord ischemia rate to our pooled analysis of open aortic procedure (46).

In light of this evidence, a number of endograft devices for the ascending aorta and aortic arch have since been developed. The GORE™ Thoracic Branch Endograft is a single-branch device designed to preserve left subclavian perfusion (47), while the Valiant Mona LSA Thoracic Endograft provides a similar off-the-shelf option for targeted revascularization (48). The Chinese Castor™ single-branched stent graft has likewise demonstrated high technical success and patency rates in thoracic aortic disease involving the left subclavian artery (49). Similar to these, The Nexus™ Endograft is a modular, single-branch system engineered for aortic arch repair that enables rapid deployment (50).

Among custom-made multibranched devices, the Terumo Relay Branch System is designed for total arch replacement (39), the Inoue™ branched stent graft has shown promising patency and stroke prevention (51) and the Zenith™ Arch Branch Graft enables multivessel revascularization with high technical success but is limited by custom manufacturing times (52).

Fenestrated and scalloped designs provide further options, including the semi-customized Najuta™ fenestrated endograft (41), the Terumo Bolton Relay scallop device for branch vessel flow (53) and the Cook Zenith™ fenestrated/scallop system (54).

### Ongoing clinical trials

Multiple trials will clarify the role of total endovascular arch repair: ARISE-II (GORE ascending stent graft), TRIOMPHE (NEXUS system), GIANT and GENIUS (WeFlow branch systems), and the UCSF branched aortic arch study. The EXTEND trial is evaluating hybrid Thoraflex™ with adjunctive RelayPro. The results of these studies will likely shift indications toward less invasive solutions and inform future guidelines. Table 1 summarized the design and endpoints of the most important current trials.

**Table 1:** Summary of ongoing clinical trials on endovascular therapy for thoracic aortic repair.

Trial (investigator)	Device(s)	Study design	Outcome measures
ARISEII (W.L. Gore & associates)	GORE^TM^ Ascending Stent Graft GORE^TM^ Thoracic Aortic Graft Branch Endoprosthesis	High operative risk patients with aortic aneurysms, pseudoaneurysms, aortic dissection, or other aortic lesions assigned to treatment with GORE^TM^ ASG +/- TBE or open repair	30-day technical success and absence of reintervention and a composite of postoperative complications at 30 days
TRIOMPHE (Endospan Ltd.)	NEXUS^TM^ Ascending Stent Graft	Chronic aortic dissection, penetration aortic ulcer and/or intramural hematoma, aortic aneurysm	30-day device failure or clinical failure
GIANT (Hangzhou Endonom Medtech Co., Ltd.)	WeFlow-Arch^TM^ Modeler Embedded Branch Stent Graft System	True/false aortic arch aneurysms and aortic arch ulcers	All-cause mortality and major postoperative stroke rates
GENIUS (Hangzhou Endonom Medtech Co., Ltd.)	WeFlow-Tribranch^TM^ Embedded Aortic Triple-branch Arch Stent Graft System	True/false aortic arch aneurysms and aortic arch ulcers	All-cause mortality and major postoperative stroke rates
UCSF branched aortic arch study (Timothy Chuter, MD)	Endovascular bifurcated stent graft	Proximal aortic aneurysms involving the aortic arch	Successful implantation of bifurcated stent graft at 1 month and stability of repair at 5 years
EXTEND (Vascutek Ltd.)	Thoraflex^TM^ Hybrid Relay^TM^ Pro Non Bare Stent	Aortic aneurysms, pseudoaneurysms, dissection, penetrating ulcers, and intramural hematomas affecting the aortic arch and descending aortia with/without involvement of the ascending aorta treated with Thoraflex^TM^ Hybrid alone and in combination with Relay^TM^ Pro NBS	Permanent disabling stroke and grade 3 spinal cord ischemia within 30 days of index procedure or first extension and at 1 year after the index procedure and 8 months after first extension All-cause mortality within 1 year of either procedure Device technical success at 1 year after the index procedure and 8 months after the first extension

ASG, ascending stent-graft; TBE, thoracic branch endoprosthesis; NBS, non bare stent.

### Limitations and gap in evidence

Despite our best efforts to provide a comprehensive summary, our review is not without its limitations. First, our pooled analysis is likely limited by heterogeneity in patient populations, arch pathology, and perioperative care, as well as a lack of comparative studies across prosthesis types.

Second, existing evidence is limited by non-randomized retrospective studies or expert opinion. Also, aortic disease constituting a small proportion of cardiovascular pathology, alongwith subdivision of patients across other subspecialties has led to underpowered studies (31). Larger scale prospective studies comparing different aortic procedures according to each individual pathology under same pre-operative, perioperative and post operative environment should be undertaken to guide individualized patient treatment.

EACTS and STS guidelines emphasize the need for improved diagnostic biomarkers, standardized follow-up and reporting, determining the extent of repair in acute type A dissection, evaluation of endovascular therapy in heritable aortic disease, and advances in understanding aortic remodeling with automated measurement of aortic dimensions (31).

## Conclusions

The frozen elephant trunk procedure has matured into a reproducible, safe, and widely adopted single-stage solution for complex arch pathology. Continuous refinement of perfusion techniques, cerebral protection, and prosthesis design continue to improve outcomes while expanding endovascular options are enabling patient-specific treatment, with open FET favored for durable repair in younger patients and less invasive approaches preferred in higher-risk groups. The integration of data from ongoing trials will be essential to define evidence-based best practices and shape the future of aortic arch surgery.

## References

[1] Forbess JM Ibla JC Lidov HG Cioffi MA Hiramatsu T Laussen P University of Wisconsin cerebroplegia in a piglet survival model of circulatory arrest. Ann Thorac Surg. (1995) 60(6 Suppl):S494–500. 10.1016/S0003-4975(21)01181-4 8604918

[2] Bozso SJ El-Andari R Nedadur R Loshusan B Smith H Chung JCY State-of-the-art review of aortic arch reconstruction with the frozen elephant trunk. Innov Phila Pa. (2025) 20(3):235–43. 10.1177/15569845251347968 PMC12265930 40589212

[3] Miyamoto Y. Elephant trunk technique for hybrid aortic arch repair. Gen Thorac Cardiovasc Surg. (2014) 62(3):135–41. 10.1007/s11748-013-0299-0 23943042

[4] Safi HJ Miller CC Estrera AL Huynh TTT Porat EE Allen BS Staged repair of extensive aortic aneurysms: long-term experience with the elephant trunk technique. Ann Surg. (2004) 240(4):677–84. discussion 684-685. 10.1097/01.sla.0000140756.30517.1b 15383795 PMC1356469

[5] Shrestha M Martens A Krüger H Maeding I Ius F Fleissner F Total aortic arch replacement with the elephant trunk technique: single-centre 30-year results. Eur J Cardio-Thorac Surg Off J Eur Assoc Cardio-Thorac Surg. (2014) 45(2):289–95. discussion 295-296. 10.1093/ejcts/ezt359 23872461

[6] Bavaria J Vallabhajosyula P Moeller P Szeto W Desai N Pochettino A. Hybrid approaches in the treatment of aortic arch aneurysms: postoperative and midterm outcomes. J Thorac Cardiovasc Surg. (2013) 145(3 Suppl):S85–90. 10.1016/j.jtcvs.2012.11.044 23260461

[7] Preventza O Aftab M Coselli JS. Hybrid techniques for complex aortic arch surgery. Tex Heart Inst J. (2013) 40(5):568–71. 24391324 PMC3853841

[8] Karck M Chavan A Hagl C Friedrich H Galanski M Haverich A. The frozen elephant trunk technique: a new treatment for thoracic aortic aneurysms. J Thorac Cardiovasc Surg. (2003) 125(6):1550–3. 10.1016/S0022-5223(03)00045-X 12830086

[9] Karck M Chavan A Khaladj N Friedrich H Hagl C Haverich A. The frozen elephant trunk technique for the treatment of extensive thoracic aortic aneurysms: operative results and follow-up. Eur J Cardiothorac Surg. (2005) 28(2):286–90. 10.1016/j.ejcts.2005.02.046 15922612

[10] Baraki H Hagl C Khaladj N Kallenbach K Weidemann J Haverich A The frozen elephant trunk technique for treatment of thoracic aortic aneurysms. Ann Thorac Surg. (2007) 83(2):S819–23. 10.1016/j.athoracsur.2006.10.083 17257934

[11] Schoenhoff FS Schmidli J Eckstein FS Berdat PA Immer FF Carrel TP. The frozen elephant trunk: an interesting hybrid endovascular-surgical technique to treat complex pathologies of the thoracic aorta. J Vasc Surg. (2007) 45(3):597–9. 10.1016/j.jvs.2006.10.038 17321346

[12] Karck M Kamiya H. Progress of the treatment for extended aortic aneurysms; is the frozen elephant trunk technique the next standard in the treatment of complex aortic disease including the arch? Eur J Cardio-Thorac Surg Off J Eur Assoc Cardio-Thorac Surg. (2008) 33(6):1007–13. 10.1016/j.ejcts.2008.02.030 18406159

[13] Acharya M Sherzad H Bashir M Mariscalco G. The frozen elephant trunk procedure: indications, outcomes and future directions. Cardiovasc Diagn Ther. (2022) 12(5):708–21. 10.21037/cdt-22-330 36329958 PMC9622409

[14] Spath P Campana F Tsilimparis N Gallitto E Pini R Faggioli G Outcomes of fenestrated and branched endografts for partial and total endovascular repair of the aortic arch – A systematic review and meta-analysis. Eur J Vasc Endovasc Surg. (2024) 67(1):106–16. 10.1016/j.ejvs.2023.07.048 37536517

[15] Benedetto U Raja SG Amrani M Pepper JR Zeinah M Tonelli E The impact of arterial cannulation strategy on operative outcomes in aortic surgery: evidence from a comprehensive meta-analysis of comparative studies on 4476 patients. J Thorac Cardiovasc Surg. (2014) 148(6):2936–.e4. 10.1016/j.jtcvs.2014.05.082 25112929

[16] Santos K Velasco EM Mawasi M Płonek T. Unilateral versus bilateral antegrade cerebral perfusion in aortic arch surgery: systematic review and meta-analysis of randomised controlled trials and propensity-matched studies. Heart Lung Circ. (2025) 34:S144395062500294X. 10.1016/j.hlc.2025.03.018 40592664

[17] Kazui T Bashar AHM. Aortic arch replacement using a trifurcated graft. Ann Thorac Surg. (2006) 81(4):1552. 10.1016/j.athoracsur.2005.08.063 16564332

[18] Spielvogel D Etz CD Silovitz D Lansman SL Griepp RB. Aortic arch replacement with a trifurcated graft. Ann Thorac Surg. (2007) 83(2):S791–5. 10.1016/j.athoracsur.2006.11.015 17257928

[19] Galvin SD Matalanis G. Continuous perfusion “branch-first” aortic arch replacement: a technical perspective. Ann Cardiothorac Surg. (2013) 2(2):22934–234. 10.3978/j.issn.2225-319x.2013.03.04 PMC3741843 23977588

[20] Kim M Matalanis G. Technique and rationale for branch-first total aortic arch repair. JTCVS Tech. (2020) 4:1–4. 10.1016/j.xjtc.2020.09.014 34317950 PMC8306982

[21] Matalanis G Koirala RS Shi WY Hayward PA McCall PR. Branch-first aortic arch replacement with no circulatory arrest or deep hypothermia. J Thorac Cardiovasc Surg. (2011) 142(4):809–15. 10.1016/j.jtcvs.2011.01.020 21329948

[22] Matalanis G Perera NK Galvin SD. Aortic arch replacement without circulatory arrest or deep hypothermia: the “branch-first” technique. J Thorac Cardiovasc Surg. (2015) 149(2, Supplement):S76–82. 10.1016/j.jtcvs.2014.07.100 25227697

[23] Tang GHL Kai M Malekan R Lansman SL Spielvogel D. Trifurcated graft replacement of the aortic arch: state of the art. J Thorac Cardiovasc Surg. (2015) 149(2):S55–8. 10.1016/j.jtcvs.2014.07.038 25173128

[24] Abt BG Bojko M Elsayed RS Han S Wang A Vu I Branch-first aortic arch replacement strategy decreases perioperative mortality. J Thorac Cardiovasc Surg. (2024) 167(6):2005–2012.e1. 10.1016/j.jtcvs.2023.08.012 37574006

[25] Camerlingo C. A Novel Modification of Frozen Elephant Trunk Technique: Unique Protocol from one Institution. Rome: European Review (2021). Available online at: https://www.europeanreview.org/article/26384 (Accessed August 17, 2025). 10.26355/eurrev_202107_26384 34337721

[26] Roselli EE Rafael A Soltesz EG Canale L Lytle BW. Simplified frozen elephant trunk repair for acute DeBakey type I dissection. J Thorac Cardiovasc Surg. (2013) 145(3):S197–201. 10.1016/j.jtcvs.2012.11.068 23260435

[27] Benedetto U Mohamed H Vitulli P Petrou M. Axillary versus femoral arterial cannulation in type A acute aortic dissection: evidence from a meta-analysis of comparative studies and adjusted risk estimates. Eur J Cardio-Thorac Surg Off J Eur Assoc Cardio-Thorac Surg. (2015) 48(6):953–9. 10.1093/ejcts/ezv035 25661080

[28] Louis L Panza A Chivasso P Mastrogiovanni G Masiello P Domenico Bruno V Home-made Branched Single Pump Circuit Simplifies Trivascular Antegrade Cerebral Perfusion in Open Aortic Arch Surgery.

[29] Preventza O Liao JL Olive JK Simpson K Critsinelis AC Price MD Neurologic complications after the frozen elephant trunk procedure: a meta-analysis of more than 3000 patients. J Thorac Cardiovasc Surg. (2020) 160(1):20–33.e4. 10.1016/j.jtcvs.2019.10.031 31757456

[30] Shrestha M Bachet J Bavaria J Carrel TP De Paulis R Di Bartolomeo R Current status and recommendations for use of the frozen elephant trunk technique: a position paper by the vascular domain of EACTS. Eur J Cardio-Thorac Surg Off J Eur Assoc Cardio-Thorac Surg. (2015) 47(5):759–69. 10.1093/ejcts/ezv085 25769463

[31] Czerny M Grabenwöger M Berger T Aboyans V Della Corte A Chen EP EACTS/STS Guidelines for Diagnosing and Treating Acute and Chronic Syndromes of the Aortic Organ-The Annals of Thoracic Surgery. Available online at: https://www.annalsthoracicsurgery.org/article/S0003-4975(24)00077-8/fulltext (Accessed September 16, 2025). 10.1016/j.athoracsur.2024.01.021 38416090

[32] Frankel WC Green SY Orozco-Sevilla V Preventza O Coselli JS. Contemporary surgical strategies for acute type A aortic dissection. Semin Thorac Cardiovasc Surg. (2020) 32(4):617–29. 10.1053/j.semtcvs.2020.06.025 32615305

[33] Riambau V Böckler D Brunkwall J Cao P Chiesa R Coppi G Editor’s choice – management of descending thoracic aorta diseases: clinical practice guidelines of the European society for vascular surgery (ESVS). Eur J Vasc Endovasc Surg. (2017) 53(1):4–52. 10.1016/j.ejvs.2016.06.005 28081802

[34] Etz CD Weigang E Hartert M Lonn L Mestres CA Di Bartolomeo R Contemporary spinal cord protection during thoracic and thoracoabdominal aortic surgery and endovascular aortic repair: a position paper of the vascular domain of the European association for cardio-thoracic surgery†. Eur J Cardio-Thorac Surg Off J Eur Assoc Cardio-Thorac Surg. (2015) 47(6):943–57. 10.1093/ejcts/ezv142 25991554

[35] Di Eusanio M Pantaleo A Murana G Pellicciari G Castrovinci S Berretta P Frozen elephant trunk surgery—the Bologna’s experience. Ann Cardiothorac Surg. (2013) 2(5):597–605. 10.3978/j.issn.2225-319x.2013.08.01 24109567 PMC3791204

[36] De Paulis R Di Bartolomeo R Murana G Di Marco L Pantaleo A Alfonsi J Frozen versus conventional elephant trunk technique: application in clinical practice. Eur J Cardiothorac Surg. (2017) 51(suppl_1):i20–8. 10.1093/ejcts/ezw335 28108565

[37] Di Eusanio M Castrovinci S Tian DH Folesani G Cefarelli M Pantaleo A Antegrade stenting of the descending thoracic aorta during DeBakey type 1 acute aortic dissection repair. Eur J Cardiothorac Surg. (2014) 45(6):967–75. 10.1093/ejcts/ezt493 24157481

[38] Pape LA Awais M Woznicki EM Suzuki T Trimarchi S Evangelista A Presentation, diagnosis, and outcomes of acute aortic dissection. JACC. (2015) 66(4):350–8. 10.1016/j.jacc.2015.05.029 26205591

[39] Jubouri M Surkhi AO Al-Tawil M Piffaretti G. Driving technology for thoracic endovascular aortic repair: an international analysis of single-versus double-branch RELAY outcomes. Ann Vasc Surg. (2023) 94:80–91. 10.1016/j.avsg.2023.02.002 36828134

[40] Nakhaei P Bashir M Jubouri M Banar S Ilkhani S Borzeshi EZ Aortic remodeling, distal stent-graft induced new entry and endoleak following frozen elephant trunk: a systematic review and meta-analysis. J Card Surg. (2022) 37(11):3848–62. 10.1111/jocs.16918 36069163

[41] Onitsuka S Tanaka A Otsuka H Shintani Y Kanamoto R Negoto S General information and applications of najuta fenestrated stent grafts for aortic arch aneurysms. J Clin Med. (2024) 14(1):36. 10.3390/jcm14010036 39797118 PMC11721579

[42] Preventza O. Commentary: one size does not fit all: the landing zone of the frozen elephant trunk will be different for every patient, and we need to be safe. J Thorac Cardiovasc Surg. (2024) 167(1):26–7. 10.1016/j.jtcvs.2022.04.030 35610070

[43] Takagi D Yamamoto H Kadohama T Kiryu K Wada T Igarashi I. Optimal stent length and distal positioning of frozen elephant trunks deployed from the aortic zone 0 for type A acute aortic dissection. J Thorac Cardiovasc Surg. (2024) 167(1):15–25.e2. 10.1016/j.jtcvs.2022.03.007 35422323

[44] Bozso SJ Nagendran J Chu MWA Kiaii B El-Hamamsy I Ouzounian M Midterm outcomes of the dissected aorta repair through stent implantation trial. Ann Thorac Surg. (2021) 111(2):463–70. 10.1016/j.athoracsur.2020.05.090 32673661

[45] Ganapathi AM Englum BR Hanna JM Schechter MA Gaca JG Hurwitz LM Frailty and Risk in Proximal Aortic Surgery. in: J Thorac Cardiovasc Surg. St. Louis, MO: Mosby Inc. (2014). p. 186–191.e1. 10.1016/j.jtcvs.2013.09.011 PMC4336171 24183336

[46] Putra RM Putranto JNE Budiarto RM Luthfah N Francio K Widiarti W. Redefining endovascular boundaries: a systematic review of physician-modified endovascular grafts in zone 2 thoracic endovascular aortic repair. Ann Vasc Surg. (2025) 121:534–42. 10.1016/j.avsg.2025.08.012 40816492

[47] DiLosa K Pozolo C Heafner T Humphries M Kwong M Maximus S. Early experience with the gore TAG thoracic branch endoprosthesis for treatment of acute aortic pathology. J Vasc Surg Cases Innov Tech. (2023) 10(1):101363. 10.1016/j.jvscit.2023.101363 38130369 PMC10731599

[48] Roselli EE Arko FR Thompson MM, Valiant Mona LSA Trial Investigators. Results of the valiant Mona LSA early feasibility study for descending thoracic aneurysms. J Vasc Surg. (2015) 62(6):1465–1471.e3. 10.1016/j.jvs.2015.07.078 26483004

[49] Ren J Chen Y E E Ma M Liu Z Zhu J Midterm outcomes of multicenter castor single-branch stent graft use in the treatment of thoracic aortic diseases. J Endovasc Ther Off J Int Soc Endovasc Spec. (2024) 27:15266028241234500. 10.1177/15266028241234500 38414233

[50] Planer D Elbaz-Greener G Mangialardi N Lindsay T D’Onofrio A Schelzig H NEXUS Arch: a multicenter study evaluating the initial experience with a novel aortic arch stent graft system. Ann Surg. (2023) 277(2):e460. 10.1097/SLA.0000000000004843 33714965

[51] Tazaki J Inoue K Higami H Higashitani N Toma M Saito N Thoracic endovascular aortic repair with branched inoue stent graft for arch aortic aneurysms. J Vasc Surg. (2017) 66(5):1340–1348.e5. 10.1016/j.jvs.2017.03.432 28583734

[52] Haulon S Greenberg RK Spear R Eagleton M Abraham C Lioupis C Global experience with an inner branched arch endograft. J Thorac Cardiovasc Surg. (2014) 148(4):1709–16. 10.1016/j.jtcvs.2014.02.072 24685375

[53] Sica S Pratesi G Rossi G Ferraresi M Lovato L Volpe P Proximal sealing in the aortic arch for inner curve disease using the custom relay scalloped and fenestrated stent graft. J Vasc Surg. (2024) 80(5):1317–1325.e2. 10.1016/j.jvs.2024.07.086 39069017

[54] Oderich GS Greenberg RK Farber M Lyden S Sanchez L Fairman R Results of the United States multicenter prospective study evaluating the zenith fenestrated endovascular graft for treatment of juxtarenal abdominal aortic aneurysms. J Vasc Surg. (2014) 60(6):1420–1428.e5. 10.1016/j.jvs.2014.08.061 25195145

