# Supplementary material for: Frozen elephant trunk: evolving techniques, persistent challenges, and the endovascular shift
Source: Front Cardiovasc Med. 2026 Jan 27;12:1716491. doi: 10.3389/fcvm.2025.1716491 (PMC12887726; doi:10.3389/fcvm.2025.1716491)
Supplement: Supplementary file 1 [file Datasheet1.docx]

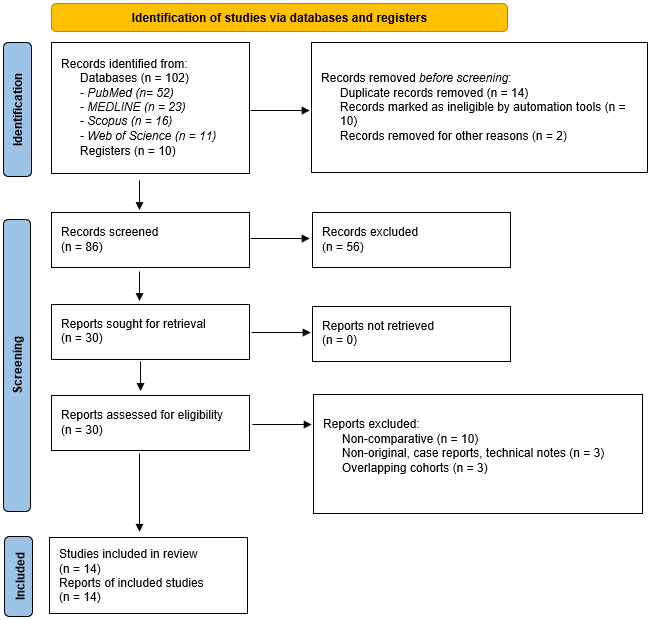


**Supplementary Figure:** PRISMA Flow Diagram

| **Study** | **Selection** | **Comparability** | **Outcome** | **Total Quality Score^+^** | **Quality Assessment** |
| --- | --- | --- | --- | --- | --- |
| Koizumi |  |  |  | 6 | Fair |
| Göbel |  |  |  | 9 | Good |
| Hage |  |  |  | 8 | Good |
| Ogino |  |  |  | 6 | Fair |
| Wisniewski |  |  |  | 8 | Good |
| Berdajs |  |  |  | 8 | Good |
| Kaneyuki |  |  |  | 8 | Good |
| Yoshitake |  |  |  | 7 | Good |
| Furutachi |  |  |  | 9 | Good |
| Inoue |  |  |  | 6 | Fair |
| Mutsuga |  |  |  | 9 | Good |
| Takagi |  |  |  | 9 | Good |
| Sirota |  |  |  | 8 | Good |
| Zhu |  |  |  | 7 | Good |

**Supplementary Table:** Quality assessment of retrospective cohort studies- Newcastle-Ottawa Scale. ^+^*Total quality score of 7-9 is considered good quality, 4-6 considered fair quality, and 0-3 considered poor quality*.
